# Supplementary material for: Altered microRNA Transcriptome in Cultured Human Liver Cells upon Infection with Ebola Virus
Source: Int J Mol Sci. 2021 Apr 6;22(7):3792. doi: 10.3390/ijms22073792 (PMC8038836; doi:10.3390/ijms22073792)
Supplement: Supplementary file 1 [file ijms-22-03792-s001.zip › Supplementary_File/C_ GO_Analysis_Results/16-30nt_go_Reston-96h-Huh7_vs_Control-96h-Huh7_down.mature_mirna_targets/BP_FoldEnrichmentDotPlot.pdf]

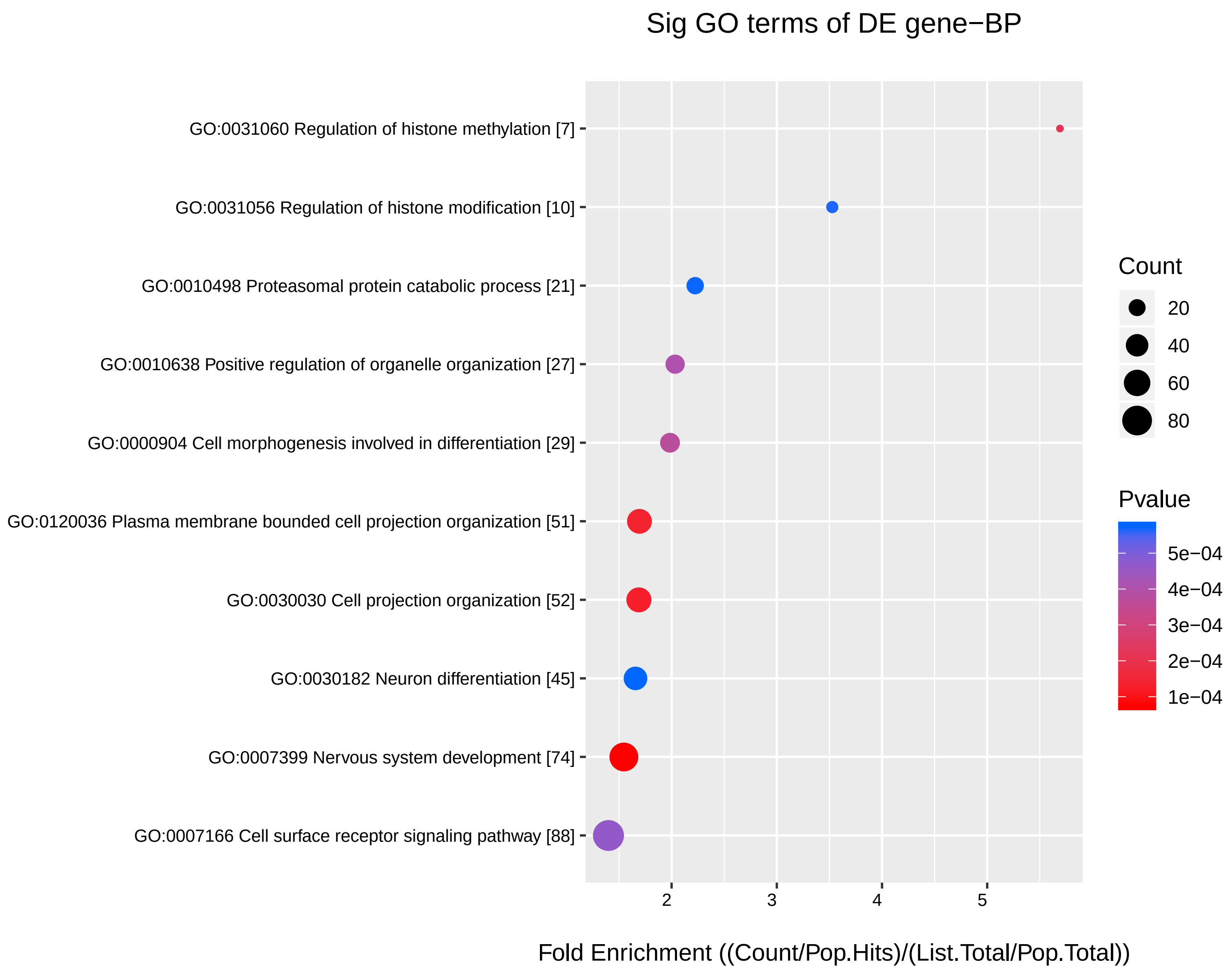

Sig GO terms of DE gene−BP
GO:0031060 Regulation of histone methylation [7]
GO:0031056 Regulation of histone modification [10]
GO:0010498 Proteasomal protein catabolic process [21]
GO:0010638 Positive regulation of organelle organization [27]
GO:0000904 Cell morphogenesis involved in differentiation [29]
GO:0120036 Plasma membrane bounded cell projection organization [51]
GO:0030030 Cell projection organization [52]
GO:0030182 Neuron differentiation [45]
GO:0007399 Nervous system development [74]
GO:0007166 Cell surface receptor signaling pathway [88]
2
3
4
5
Fold Enrichment ((Count/Pop.Hits)/(List.Total/Pop.Total))
Count
20
40
60
80
Pvalue
5e−04
4e−04
3e−04
2e−04
1e−04
